# Multifractal and Chaotic Properties of Solar Wind at MHD and Kinetic Domains: An Empirical Mode Decomposition Approach

**DOI:** 10.3390/e21030320

**Published:** 2019-03-25

**Authors:** Tommaso Alberti, Giuseppe Consolini, Vincenzo Carbone, Emiliya Yordanova, Maria Federica Marcucci, Paola De Michelis

**Affiliations:** 1 INAF-Istituto di Astrofisica e Planetologia Spaziali, via del Fosso del Cavaliere 100, 00133 Rome, Italy; 2 Dipartimento di Fisica, Università della Calabria, Ponte P. Bucci, 87036 Rende, Italy; 3 Swedish Institute of Space Physics, 75121 Uppsala, Sweden; 4 Istituto Nazionale di Geofisica e Vulcanologia, via di Vigna Murata 605, 00143 Rome, Italy

**Keywords:** solar wind, scaling properties, fractals, chaos

## Abstract

Turbulence, intermittency, and self-organized structures in space plasmas can be investigated by using a multifractal formalism mostly based on the canonical structure function analysis with fixed constraints about stationarity, linearity, and scales. Here, the Empirical Mode Decomposition (EMD) method is firstly used to investigate timescale fluctuations of the solar wind magnetic field components; then, by exploiting the local properties of fluctuations, the structure function analysis is used to gain insights into the scaling properties of both inertial and kinetic/dissipative ranges. Results show that while the inertial range dynamics can be described in a multifractal framework, characterizing an unstable fixed point of the system, the kinetic/dissipative range dynamics is well described by using a monofractal approach, because it is a stable fixed point of the system, unless it has a higher degree of complexity and chaos.

## 1. Introduction

The interplanetary space is permeated by a supersonic and super-Alfvénic plasma known as solar wind which develops a strong turbulent character during its expansion phase [1]. Due to the presence of a mean magnetic field, solar wind low-frequency fluctuations are usually described within the magnetohydrodynamic (MHD) framework [2,3,4]. These fluctuations show turbulent properties that are characterized by a quasi-Kolmogorov energy scaling [5,6,7,8]. Indeed, magnetic energy density seems to follow a spectral decay as $E\left(k\right)∼k^{-5/3}$, although the theoretical scaling derived from MHD equations suggests a slightly different spectral exponent, e.g., $E\left(k\right)∼k^{-3/2}$ [9,10] for Alfvènic turbulence as it should be in the case of solar wind. Turbulence is a phenomenon showing the presence of small scale fluctuations in the velocity and pressure fields (for fluids), as well as in the magnetic field (for plasmas), and an increased rate of mixing of mass and momentum [1,11]. Turbulent flows exhibit characteristic phenomena like coherent structures in the flow and intermittency. Coherent structures are usually defined as regions of concentrated vorticity where phase correlation exists with a typical lifetime larger than that of the stochastic fluctuations surrounding them, while intermittency is the manifestation of sudden field changes, modifying the shape of the probability distribution functions of field gradients (e.g., velocity and temperature in fluids, magnetic in plasmas) [12,13], and resulting in an anomalous scaling of the field increments [14]. As the analytic and numerical solution of such flows is expensive, investigators rely on models to simulate and simplify their dynamics. Such turbulence models include two-equation models (like the *k*-ϵ model and the *k*-ω one [15]), Reynolds stress models (like the Speziale-Sarkar-Gatski model [16] and the Mishra-Girimaji model [17]), along with models in Large Eddy Simulations [18]. Similar models have been also developed for describing turbulent features in plasmas like two-dimensional hybrid-Vlasov simulations [19], compressible Hall MHD direct numerical simulations [20], and shell models [21].

In the framework of turbulence, several phenomena inside the MHD/inertial domain are described, by using the nonlinear energy cascade via the Yaglom law, an exact relation for the scaling of the third-order moment of fluctuations [22,23], or by analyzing the role of the intermittency in changing scaling properties of magnetic fluctuations within a multifractal approach [14,24,25,26]. Both previous findings are derived from the structure function analysis, through which scaling laws can be investigated (e.g., [27]), exploiting Kolmogorov’s universality assumptions (e.g., [28]). More specifically, a turbulent flow is sustained by a persistent source of energy which is rapidly dissipated via the so-called nonlinear energy cascade [29], converting the kinetic energy into internal energy through viscous processes. Indeed, turbulence causes the formation of eddies at different scales and energy is transferred from large- to small-scale structures through an inertial and inviscid mechanism [5,28], i.e., the nonlinear energy cascade. According to Kolmogorov’s theory, if all possible symmetries of the Navier-Stokes equation are restored in a statistical sense, then the turbulent flow is self-similar at small scales and has a finite mean energy dissipation rate ϵ such that, at very high (but not infinite) Reynolds numbers, all small-scale statistical properties are uniquely and universally determined by the length scale ℓ, the mean energy dissipation rate ϵ, and the kinematic viscosity ν (e.g., [28]). By simply exploiting dimensional arguments, these assumptions imply that the energy spectrum at large wavenumbers assumes a universal form as

(1) $$E\left(k\right)=F\left(\nu \right)\epsilon^{2/3}k^{-5/3}.$$

In the limit of infinite Reynolds numbers, Equation (1) becomes independent by the viscosity ν such that

(2) $$E\left(k\right)=C\;\epsilon^{2/3}k^{-5/3}.$$

being *C* a universal dimensionless constant [28]. The above assumptions are only valid for all those scales which are smaller than the integral scale *L*, where long-range correlations between particles are found, and are greater than the dissipative scale $\ell_{D}$, where viscosity dominates and the turbulent kinetic energy is dissipated, i.e., $\ell_{D}\ll \ell \ll L$ (e.g., [5,28]). Particularly, the dissipation of kinetic energy mostly takes place at the so-called Kolmogorov microscale defined as

(3) $$\eta ={\left(\frac{\nu^{3}}{\epsilon }\right)}^{1/4}$$

which is well separated from the integral scale *L*, corresponding to the size of the eddies when they are formed. These two scales mark the extrema of the energy cascade: since eddies with size *L* are much larger than the dissipative eddies with size η, kinetic energy is not dissipated at large scales but it is essentially transferred to smaller scales where viscous effects become dominant. Within this range, where nonlinear interactions between eddies take place, inertial effects are larger than viscous ones such that it is usually named “inertial range”. Due to the large separation between *L* and η, the dissipation rate is primarily determined by the large scales since viscous effects at Kolmogorov scales rapidly dissipate energy. Thus, the overall rate of dissipation is only controlled by the nonlinear scale-to-scale transfer such that the dissipation rate is approximately given as

(4) $$\epsilon =\frac{u^{3}}{L}$$

being *u* the bulk velocity of the flow, and consequently

(5) $$\frac{\eta }{L}=Re^{-3/4}$$

where Re is the Reynolds number. Equation (5) can be used as a measure of the number of scales within the inertial range (i.e., the extension of this range of scales) which only depends by the Reynolds number of the flow.

Similar assumptions and scalings can be also derived by using MHD equations for describing plasma dynamics and, particularly, within the same Kolmogorov’s assumptions of isotropy, homogeneity, and stationarity, and exploiting dimensional arguments, the energy spectrum at large wavenumbers behaves as

(6) $$E\left(k\right)=C\;{\left(\epsilon v_{0}\right)}^{1/2}k^{-3/2}.$$

where *C* a universal dimensionless constant and $v_{0}$ is the rms component of the total turbulent velocity [9,10].

Kolmogorov assumptions break down beyond a scale $\ell_{b}∼\ell_{i}$, being $\ell_{i}$ the ion inertial length, where the MHD/inertial description cannot be used [30,31], and for which a steeper slope of the energy density spectrum is found [32]. The second power-law domain at small scales can be explained by invoking several possible dispersive/kinetic phenomena as wave-wave coupling, Landau damping, Kinetic Alfvén Waves (KAWs), and so on [33,34,35].

Solar wind magnetic field fluctuations, including spectral features like the Kolmogorov energy scaling, are usually investigated by following a statistical approach of magnetic increments

(7) δb(x,ℓ)≐b(x,ℓ)−b(x)

requiring that the statistics of these increments is invariant under arbitrary translations

(8) 〈δb(x+r,ℓ)〉=〈δb(x,ℓ)〉.

In this way, it is possible to derive an exact law for the scaling of the mean-square magnetic field increments between two points separated by a distance ℓ according to which $\langle {\left(\delta b\left(\ell \right)\right)}^{2}\rangle \;∼\;\ell^{1/2}$, from which the Iroshnikov-Kraichnan $k^{-3/2}$ scaling law can be simply derived by noting that $\langle {\left(\delta b\left(\ell \right)\right)}^{2}\rangle$ is related to the magnetic energy [9,10]. Moreover, from dimensional analysis it is easy to recover the second-order structure function which is equal to $S_{2}\left(l\right)=\langle {\left(\delta b\left(\ell \right)\right)}^{2}\rangle =C_{IK}{\left(\epsilon \;c_{A}\right)}^{1/2}\ell^{1/2}$, where $c_{A}$ is the Alfvén speed and $C_{IK}$ is a universal constant. More generally, generalized structure functions can be calculated from any arbitrary and finite order *q* from increments such that

(9) $$S_{q}\left(\ell \right)≐\langle |\delta b\left(x,\ell \right){|}^{q}\rangle .$$

They are extensively used to investigate scaling properties of both velocity and magnetic field fluctuations (e.g., [27]), mostly devoted to the characterization of intermittency and self-similarity properties of solar wind turbulence [14,36,37,38]. In this context, different multifractal models (e.g., β-model, *p*-model, and their variations (e.g., [24,26])) have been proposed to explain the evolution of intermittency across the heliosphere, with the solar wind becoming more multifractal in nature when leaving the Sun [1]. In particular, the analysis of scaling exponents ζ(q) of the q-th order structure function shows that different turbulent scenarios can develop, being mainly characterized by a nonlinear dependence of ζ(q) with *q* (e.g., [39,40,41,42]).

This paper approaches the study of the self-similarity properties of solar wind magnetic field fluctuations at different timescales by using a novel method to evaluate structure functions at different orders *q*. It is based on the Empirical Mode Decomposition (EMD) which allows to correctly derive the timescales embedded into the analyzed time series, as well as to better evaluate structure functions by using local (non-constant) timescales [43,44]. The results evidence a different behavior of magnetic field fluctuations at MHD/inertial and kinetic/dissipative scales. While the former are characterized by a multifractal character, the latter show a monofractal scaling. In a dynamical system framework, both behaviors can be seen as corresponding to two different fixed points: an unstable saddle for the MHD/inertial domain, and a stable node for the dissipative one. The solar wind magnetic field fluctuations undergo a saddle-node bifurcation when moving from the MHD/inertial down to kinetic/dissipative scales.

## 2. Data

We consider solar wind magnetic field measurements from ESA-Cluster mission on 10 January 2004 from 05:30 UT to 06:30 UT. This period is characterized by a high speed (i.e., it is a fast stream, v∼ 540 km/s), a mean magnetic field intensity (B∼ 11 nT), and a high density (n∼ 14 cm${}^{-3}$). We used combined magnetic field data from the fluxgate magnetometer (FGM) and the experiment called “spatiotemporal analysis of field fluctuations” (STAFF) onboard Cluster 3 spacecraft to obtain a resolution of data equals to 450 Hz. For computational purposes, the time resolution has been reduced of a factor 4, moving it to Δt = 8.9 ms.

**Figure 1:**
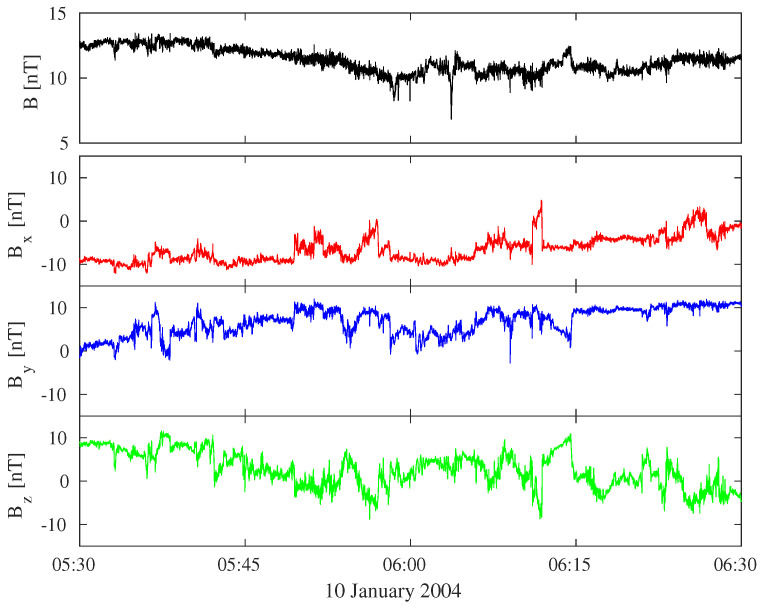
Solar wind magnetic field measurements during the time interval 05:30–06:30 UT on 10 January 2004. Data are obtained from Cluster 3 at the time resolution of 8.9 ms.

Figure 1 shows the magnetic field intensity (B) and the three magnetic field components ($B_{x},B_{y},B_{z}$) for the selected time interval in the GSE reference system.

## 3. Methods

### 3.1. The Empirical Mode Decomposition (EMD): A Brief History

During the past decades, several decomposition procedures have been suggested to investigate scale variability of time series. Most common methods rely on Fourier-based techniques, Wavelet transforms and/or eigenfunction analysis (e.g., [45]). These methods, by choosing a decomposition basis in a mathematical space with requirements of completeness and orthogonality, allow to obtain oscillating components, with fixed scales and amplitudes, embedded inside time series (e.g., [45]). However, neither stationarity nor linearity is assured when natural phenomena are investigated, unless scaling law theory is mostly derived by exploiting these two requirements (e.g., [27,28]). Recently, the Empirical Mode Decomposition (EMD) has been developed to provide a suitable decomposition method for time series by exploiting their local properties, allowing us to reduce mathematical assumptions by using a completely adaptive and a posteriori decomposition procedure where the number of the extracted empirical modes depends on the signal complexity [43]. The EMD carries out a finite set of embedded modes, usually named Intrinsic Mode Functions (IMFs), from a given time series x(t) by using an iterative process known as sifting process. The main steps of this process can be summarized as follows:

1. evaluate the mean of a signal x(t) and subtract from it to produce a zero-mean signal $x_{m}\left(t\right)\;=\;x\left(t\right)\;-\;\langle \;x\left(t\right)\;\rangle$;
2. find local maxima and minima of $x_{m}\left(t\right)$;
3. use a cubic spline to evaluate the upper ($e_{max}\left(t\right)$) and lower ($e_{min}\left(t\right)$) envelopes from local maxima and minima, respectively;
4. evaluate the mean envelope $e_{m}\left(t\right)$ and subtract from $x_{m}\left(t\right)$ to have $h\left(t\right)=x_{m}\left(t\right)-e_{m}\left(t\right)$;
5. check if h(t), often called detail or “candidate” IMF, is an IMF that is, check if the number of zero crossings and local extrema differs at most by one and if the local mean is zero;
6. if h(t) is an IMF, then store it ($c_{k}\left(t\right)=h\left(t\right)$), else repeat steps from 1 to 5 on the signal $x_{h}\left(t\right)=x_{m}\left(t\right)-h\left(t\right)$ until an IMF is obtained.

Once the decomposition is complete, i.e., when no more IMFs can be extracted from x(t), the time series x(t) can be written as

(10) $$x\left(t\right)=\sum_{k=1}^{N}c_{k}\left(t\right)+r\left(t\right)$$

where r(t) is the residue of the decomposition, a non-oscillating function of time [43]. Mathematically, the sifting process stops only when the number of iterations n→∞; numerically, it can be stopped after $n^{\prime }$ iterations by defining a stopping criterion [46] like the Cauchy convergence criterion [43], according to which the sifting algorithm stops when $\sigma_{n^{\prime }}<\sigma_{0}$, being $\sigma_{n^{\prime }}=\sum_{j=1}^{T}\frac{|h_{n^{\prime }}\left(t_{j}\right)-h_{n^{\prime }-1}\left(t_{j}\right){|}^{2}}{h_{n^{\prime }-1}^{2}\left(t_{j}\right)}$, where $h_{n^{\prime }}$ is the $n^{\prime }$ detail and *T* the length of the time series x(t), and $\sigma_{0}$ is a threshold value which usually varies between 0.2 and 0.3 [43], or by the threshold method proposed in Reference [47] in which two thresholds, $\theta_{1}$ and $\theta_{2}$, are chosen to guarantee globally small fluctuations and, in the meanwhile, to take into account locally large excursions. In this way, by defining $\sigma \left(t\right)=\left|\frac{2\;h_{n^{\prime }}\left(t\right)}{e_{max}\left(t\right)-e_{min}\left(t\right)}\right|$, the sifting process is iterated until $\sigma \left(t\right)<\theta_{1}$ for a prescribed fraction 1−α of the total duration, and $\sigma \left(t\right)<\theta_{2}$ for the remaining fraction, being typically $\theta_{1}=0.05$ and $\theta_{2}=10\;\theta_{1}$ [47,48]. More details about the sifting process and its features can be found in several previous works (e.g., [43,47,48,49]).

The EMD is a fundamental step for providing non-stationary oscillating components which can be used as inputs for the Hilbert Spectral Analysis (HSA), which permits us to investigate amplitude-frequency modulation embedded in time series (e.g., [43,50]). Through the Hilbert Transform (HT), which is a linear mathematical operator that takes each IMF $c_{k}\left(t\right)$ and produces a function $H\left[c_{k}\right]\left(t\right)$ by convolution with the function $\frac{1}{\pi t}$, each empirical mode can be written as modulated both in amplitude and in frequency

(11) $$c_{k}\left(t\right)=a_{k}\left(t\right)ℜ\left\{exp\left[i2\pi \int_{0}^{t}f_{k}\left(t^{\prime }\right)dt^{\prime }\right]\right\}$$

where $a_{k}\left(t\right)$ and $f_{k}\left(t\right)$ are the instantaneous amplitude and frequency of the *k*-th empirical mode, respectively, and ℜ is the real part of the exponential. The HT allows to investigate non-stationary features of the time series, being $f_{k}\left(t\right)$ a function of time, and also its nonlinear behavior, due to the time-dependence of $a_{k}\left(t\right)$ (e.g., [43,51]). Derived from both $a_{k}\left(t\right)$ and $f_{k}\left(t\right)$, the instantaneous local energy content E(t,f) is studied by contouring the squared-amplitude in a time-frequency plane, i.e., by defining the so-called Hilbert-Huang spectrum [43]. Then, an intermittency measure, similar to that defined by using wavelet analysis, can be introduced as

(12) $$DS\left(f\right)=\frac{1}{n\Delta t}\int_{t}{\left[1-\frac{H(t^{\prime },f)}{h(f)}\right]}^{2}dt^{\prime }$$

where $h\left(f\right)={\langle H\left(t^{\prime },f\right)\rangle }_{t}$ and nΔt is the length of the time series (e.g., [43]). It is often called Degree of Stationarity (DS) (e.g., [43]) and a time series is statistically stationary if DS = 1.

**Figure 2:**
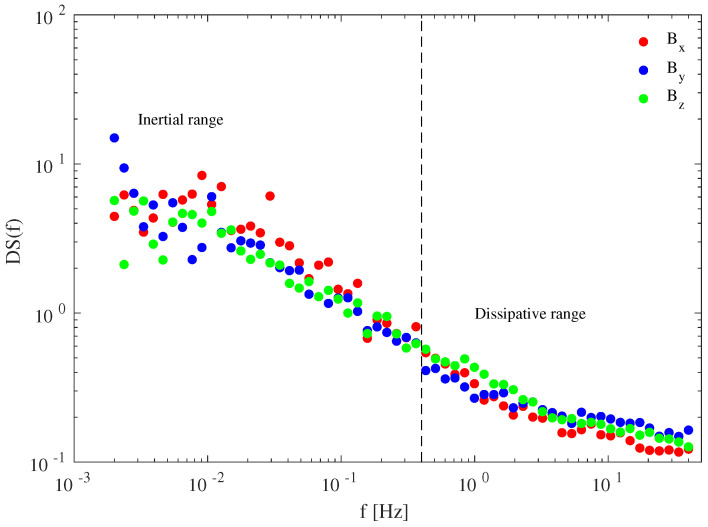
Degree of stationarity (DS) of the three different magnetic field components during the selected time interval. The dashed line refers to the Doppler-shifted ion cyclotron frequency ($f_{i}\;∼\;0.4$ Hz).

Figure 2 reports the degree of stationarity for the three magnetic field components. A clear increase in the stationary character of time series is found when approaching the frequency $f_{b}=$ 0.4 Hz $∼f_{i}$, being $f_{i}$ the Doppler-shifted ion cyclotron frequency. This suggests that a high non-stationary behavior characterizes the inertial regime, where MHD processes govern the dynamics of the system, while dissipative processes are characterized by a nearly-stationary dynamics as also previously observed (e.g., [52]). The non-stationary character observed in the MHD/inertial domain could be a counterpart of the intermittent nature of fluctuations in the inertial range.

### 3.2. The EMD-Based Multifractal Analysis

Recently, a method capable of detecting the fractal dimension of a time series by partitioning the time and scale domain of a signal into fractal dimension regions has been proposed. This method, which is similar to the Wavelet Transform Modulus Maxima (WTMM), is an EMD-based multifractal analysis. It is named EMD-based dominant amplitude multifractal formalism (DAMF) [44] and it has been proposed to investigate singularities and (multi)fractal behavior of time series. The EMD-DAMF method can be summarized in the following steps:

1. derive instantaneous amplitude $a_{k}\left(t\right)$ and mean timescale $\tau_{k}={\langle f_{k}\left(t\right)\rangle }_{t}^{-1}$ of each empirical mode;
2. determine the dominant amplitude coefficients $u_{j,k}$ over a time support $I_{j,k}$ around the *j*-th local maximum
  (13) $$u_{j,k}≐\underset{k^{\prime }\leq k}{\sup}\left\{\max\left\{|a_{k^{\prime }}\left(t\in I_{j,k}\right)|\right\}\right\}$$
  with $j=1,\cdots ,N_{k}$, being $N_{k}$ the number of local maxima of $a_{k}\left(t\right)$, and k=1,⋯,N;
3. evaluate the *q*-th-order structure function $S_{q}\left(\tau_{k}\right)$
  (14) $$S_{q}\left(\tau_{k}\right)=\frac{1}{N_{k}}\sum_{j=1}^{N_{k}}{\left\{u_{j,k}\right\}}^{q};$$
4. estimate the scaling exponent ζ(q) as the linear slope, in a log-log space, of $S_{q}\left(\tau_{k}\right)$ vs. $\tau_{k}$, such that
  (15) $$S_{q}\left(\tau_{k}\right)∼\tau_{k}^{\zeta (q)};$$
5. derive the singularity strengths α and spectrum f(α) by using the Legendre transform of the scaling exponents ζ(q) as usual
  (16) $$\alpha =\frac{d\zeta (q)}{dq}\;\&\;f\left(\alpha \right)=\alpha q-\zeta \left(q\right).$$

The main novelty introduced by this method is that structure functions $S_{q}\left(\tau_{k}\right)$ are derived by exploiting the local features of empirical modes such that local extrema can be used to correctly calculate differences/increments between two points, instead of considering a fixed timescale as for canonical structure function analysis. Moreover, timescales are not fixed a priori but they are derived from the EMD analysis of time series considering a finite set of multiresolution coefficients $u_{j,k}$. This allows us to have a limited (and small) number of points in the scaling range such that the scaling exponents can be better evaluated and visually inspected.

## 4. Results from the EMD-Based Multifractal Analysis

**Figure 3:**
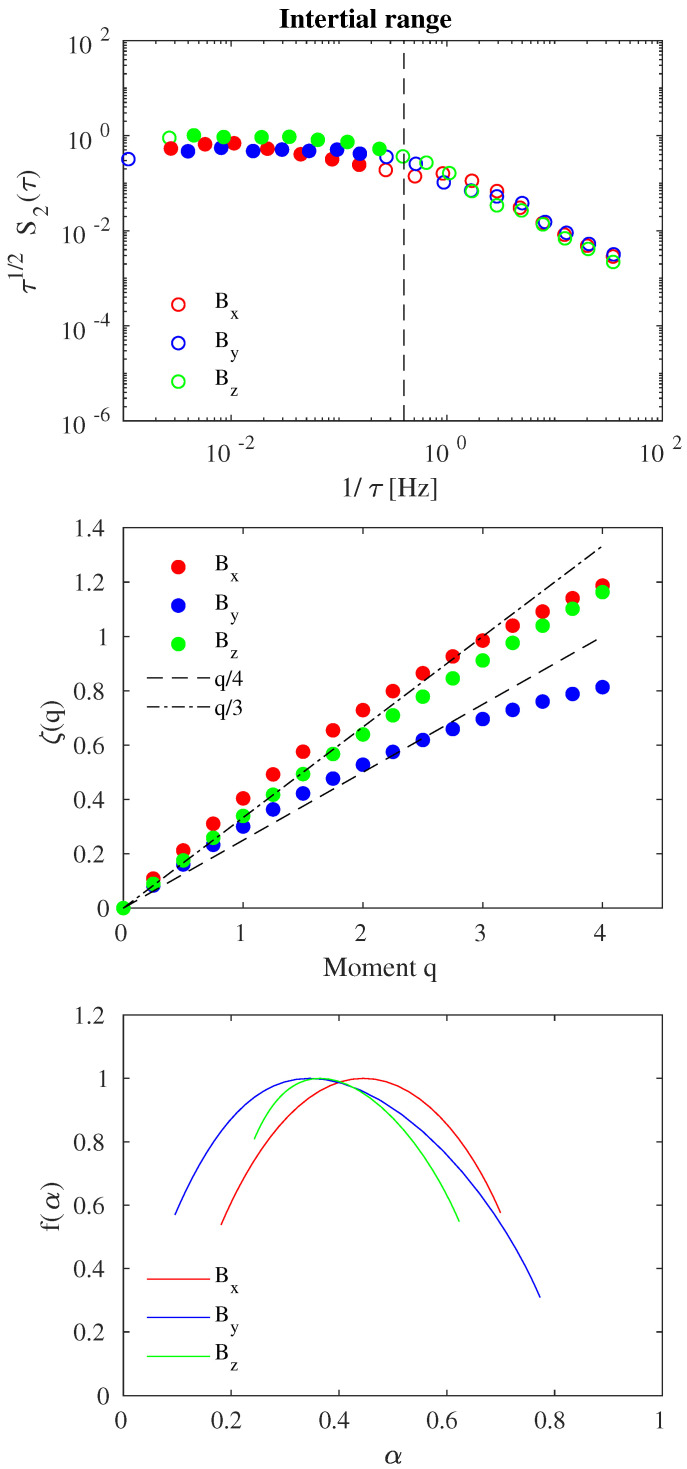
Empirical Mode Decomposition-Dominant Amplitude Multifractal Formalism (EMD-DAMF) results for the inertial range: compensated second-order structure function $S_{2}\left(\tau \right)$ (**upper panel**), scaling exponents ζ(q) (**middle panel**), and singularity spectrum f(α) (**lower panel**). Red, blue and green symbols refer to the $B_{x},B_{y},$ and $B_{z}$ solar wind magnetic field components, respectively. Filled symbols in the upper panel refer to the magnetohydrodynamic (MHD)/inertial scales where a clear Iroshnikov-Kraichnan (IK) spectrum is found. The dashed and dashed-dotted lines in the middle panel refer to ζ(q)=q/4 and ζ(q)=q/3, respectively.

**Figure 4:**
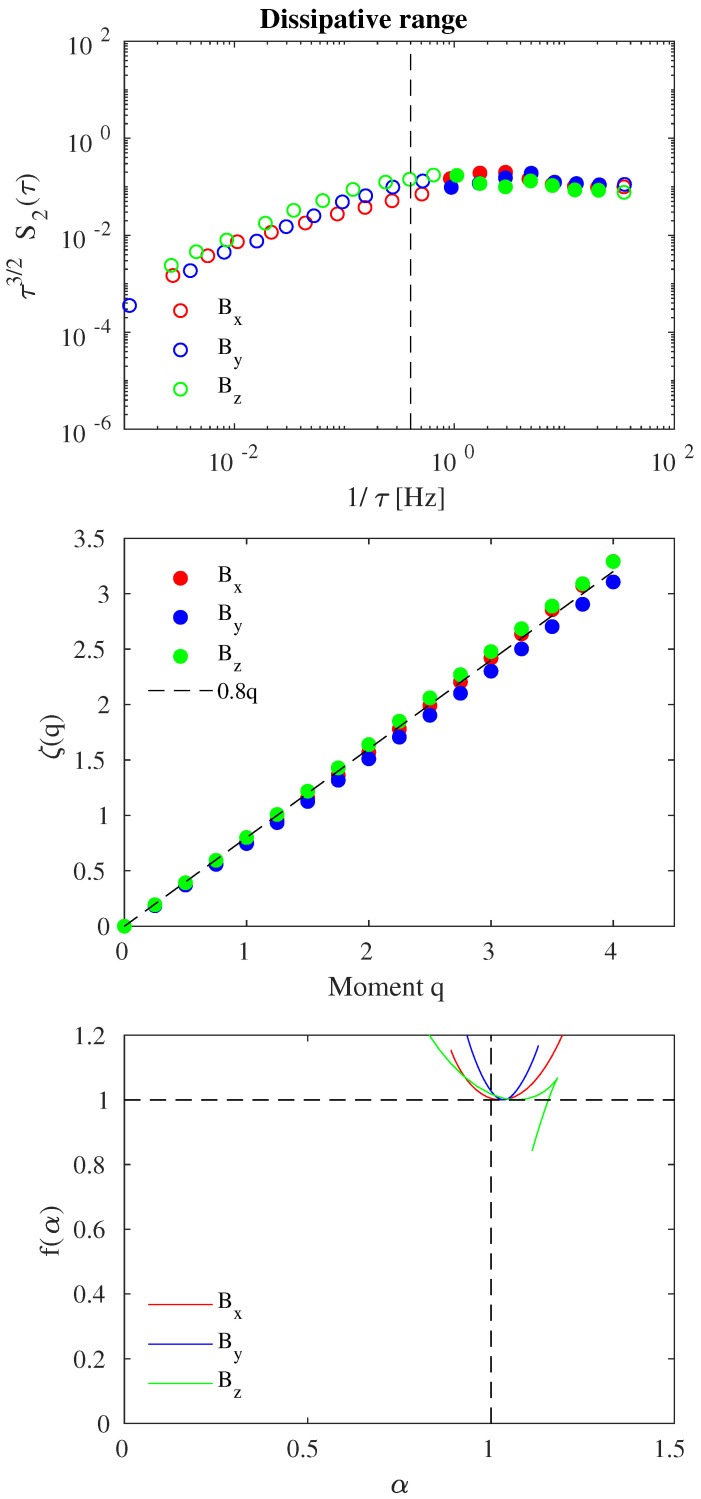
EMD-DAMF results for the dissipative range: compensated second-order structure function $S_{2}\left(\tau \right)$ (**upper panel**), scaling exponents ζ(q) (**middle panel**), and singularity spectrum f(α) (**lower panel**). Red, blue and green symbols refer to the $B_{x},B_{y},$ and $B_{z}$ solar wind magnetic field components, respectively. Filled symbols in the upper panel refer to the kinetic/dissipative scales. The dashed line in the middle panel refers to ζ(q)=0.8q.

Figure 3 and Figure 4 report the EMD-DAMF results at MHD/inertial and kinetic/dissipative scales for each magnetic field component, respectively. In each figure, the second-order structure function $S_{2}\left(\tau \right)$ is shown in the upper panel (multiplied by $\tau^{1/2}$ and $\tau^{3/2}$ to have a compensated structure function), the scaling exponents ζ(q) are reported in the middle panel, and the singularity spectrum f(α) is displayed in the lower panel.

The EMD-DAMF analysis at MHD/inertial scales (Figure 3), i.e., corresponding to the inertial range, can be carried out by considering empirical modes with mean timescales in the range 2–500 s (or $f\in (10^{-3},0.4)$ Hz). Indeed, as shown by the second-order structure function $S_{2}\left(\tau \right)$ a scale-break is found when $f=f_{b}=$ 0.4 Hz $∼f_{i}$. As expected from structure function theory of the MHD/inertial domain (e.g., [9,10]), the second-order structure function behaves as $\tau^{1/2}$, suggesting that the Fourier energy spectral density decays as $f^{-3/2}$ (or $k^{-3/2}$ assuming Taylor’s hypothesis) (e.g., [8,9,10]).

This result supports the common view according to which energy is injected at large scales (i.e., larger than a typical injection scale *L*) and transferred to small scales (i.e., smaller than a dissipative scale $\ell_{D}$) through nonlinear interactions and phenomena taking place at scales ℓ, being $\ell_{D}\ll \ell \ll L$ (e.g., [3,4,24,26]). Here, $\ell_{D}$ stands for the dissipation scale (equivalent to Kolmogorov’s scale in fluid turbulence). This result has been obtained by considering the “true” timescales which are embedded in the raw time series and extracted via an adaptive procedure, with no assumptions on the stationarity of oscillating components.

By considering structure functions $S_{q}\left(\tau \right)$ with 2 s <τ< 500 s, the scaling exponents are derived and shown in the middle panel of Figure 3 for the three magnetic field components. From a theoretical point of view, assuming homogeneity, isotropy and scale-invariance of the time series we should obtain ζ(q)=q/4 in the case of Alfvènic MHD turbulence [10]. Our results show a different behavior with scaling exponents characterized by a nonlinear convex trend with the moment order *q* like ζ(q)∼q/4+φ(q/4) [26]. This deviation is the fingerprint of the occurrence of anomalous scaling features, i.e., of an intermittency phenomenon, in the nonlinear energy cascade of the magnetic field, suggesting nonlinear two-point correlations in the real space [39,40,41]. Interestingly, different scaling exponents are obtained for the different magnetic field components indicating the existence of an anisotropy of the scaling features in the different directions which may reflect the anisotropic nature of the fluctuation field [7]. Moreover, the observed nonlinear scaling of ζ(q) suggests that the probability distribution functions (PDFs) of increments at MHD/inertial scales are characterized by multifractal scaling features. This aspect can be clearly seen by looking at the singularity spectrum f(α) (see Figure 3, lower panel) which shows a wide range of singularities (0.1 <α< 0.8) for all components. Wider singularities are found for the $B_{y}$ component, while a narrower spectrum is found for $B_{z}$.

A clear different behavior is found when approaching the dissipative range (see Figure 4), i.e., moving towards higher frequencies ($f>f_{b}=0.4$ Hz). A different scaling law is recovered, moving to a greater scaling exponent ($\tau^{3/2})$ and, consequently, a steeper slope for the energy spectral density, decaying as $∼f^{-5/2}$, is recovered. This suggests that different physical processes operate inside this dynamical regime occurring on small scales. From a fractal point of view, magnetic field fluctuations at kinetic/dissipative scales seem to behave as a monofractal system with a Hurst exponent (i.e., ζ(1)) ∼ 0.8 [52,53], quite similar for all magnetic field components (Figure 4, middle panel). This behavior is confirmed by the singularity spectrum f(α) (see Figure 4, lower panel) which collapses near the point (α,f(α))=(1,1). Our findings suggest the absence of intermittency at dissipative scales, which is well in agreement with previous works where generalized Hilbert spectra were used [52,53,54].

The difference in the scaling properties between the two ranges of scales can be linked to the different physical processes operating in both the inertial and dissipative domains. On one hand, the inertial range is characterized by the nonlinear interactions between eddies of different size, causing their fragmentation to smaller and smaller ones until viscous effects become dominant (it is worthwhile to remark that eddies must not be thought of as real vortices, but as a description of the triadic interaction between modes). Conversely, when approaching the Kolmogorov scale η wave-particle mechanisms and small-scale structures (like current sheets) become the most prominent features which characterize the dissipative processes [31]. Indeed, while the inertial range physics is mostly dominated by large-scale phenomena like plasma instabilities and it is characterized by an inhomogeneous nonlinear transfer of energy, resulting in the generation of localized small-scale structures with scale-dependent features [8,14,36], the dissipative range physics is mainly characterized by several dispersive phenomena generated by velocity-space effects and electron dynamics, driven by wave-wave coupling, scattering processes, and damping mechanisms [1,31,34,35].

Moreover, our results seem to confirm the robustness of the EMD-based method in investigating scaling features of solar wind fluctuations. In addition, by using the EMD-DAMF approach we are able to carry out structure function analysis on both positive and negative *q*, allowing us to derive the whole singularity spectrum f(α) such that accurate intermittency measures can be found. Conversely, generalized Hilbert spectra, unless based on the EMD and HSA procedures, cannot be evaluated for q<0, thus permitting only a partial detection of singularities (only the increasing branch of f(α) can be obtained) [55]. Although the difference in the intermittent properties between MHD/inertial and kinetic/dissipative domains remains an open question (e.g., [35]), our results can help to accurately measure scaling exponents and singularities with fewer a priori mathematical assumptions with respect to previous analysis, thus providing useful constraints for modeling purposes.

## 5. Chaotic Measures and Phase-Space Analysis

A dynamical system, like the solar wind, can be also investigated following a chaotic approach, mostly based on looking at the dimensionality of its phase-space. A system is defined to be chaotic if its dimension is a non-integer value [56]. Different measures have been introduced to quantify the presence and degree of chaos [57]. Particularly, the correlation dimension $D_{2}$, useful for determining the fractional dimensions of fractal objects, is estimated by embedding a time series x(t) in a time-delayed *m*-component state vector as

(17) $$X_{k}=\left\{x_{1}\left(t_{k}\right),x_{2}\left(t_{k}\right),\cdots ,x_{m}\left(t_{k}\right)\right\}$$

where $x_{l}\left(t_{k}\right)=x\left(t_{k}+\left(l+1\right)\Delta \right)$, *m* is usually named embedding dimension, and Δ is a time delay. Then, the correlation integral can defined as

(18) $$C\left(\rho ,m\right)=\lim_{N_{s}\to \infty }\frac{1}{N_{s}^{2}}\sum_{i=1}^{N_{s}}\sum_{j=1}^{N_{s}}\Theta (\rho -|X_{i}-X_{j}\left|\right)$$

where $N_{s}$ is the number of considered phase-space states, Θ is the Heavyside step function, and ρ is the phase-space threshold distance between two points. If ρ→0, a power-law behavior is found for the correlation integral as $C\left(\rho ,m\right)∼\rho^{D_{2}}$, where $D_{2}$ is defined as

(19) $$D_{2}=\lim_{\rho \to 0}\frac{\log C(\rho ,m)}{\log\rho }.$$

As the embedding dimension *m* increases, the correlation dimension will converge to its true value. Specifically, if $D_{2}=m$ then the system will explore the whole phase-space; conversely, if $D_{2}<m$ a strange attractor will characterize the phase-space dynamics. Of course, both *m* and Δ need to be properly chosen. Their choice is crucial for a correct estimation of the correlation dimension in the case of chaotic systems [56,57,58]. Generally, the choice of the time delay Δ corresponds to the first minimum of the autocorrelation function of the time series, while the choice of the embedding dimension *m* falls on the lowest value at which $D_{2}$ approaches from a constant value [57].

**Figure 5:**
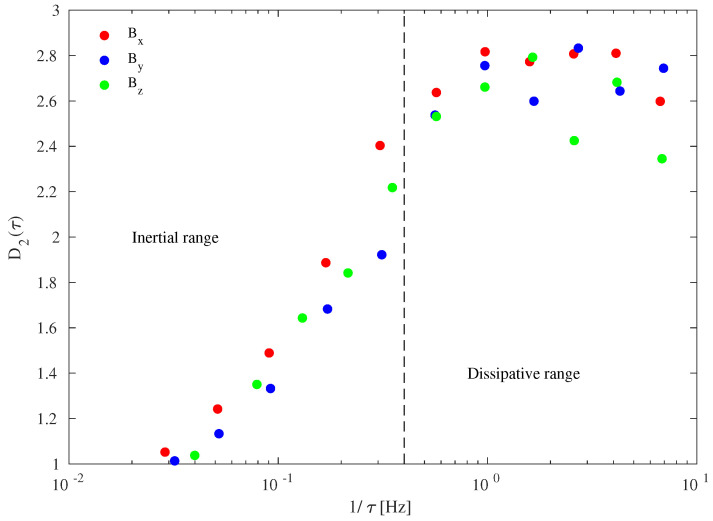
Correlation dimension $D_{2}$ of the different empirical modes as function of the mean frequency (1/τ). The vertical dashed line separates the inertial range from the kinetic/dissipative one.

Figure 5 shows the behavior of the correlation dimension $D_{2}\left(\tau \right)$ as a function of the mean frequency of each empirical mode, derived as the inverse of the mean timescale τ. This allows us to investigate how the dynamical behavior changes when moving from MHD/inertial to kinetic/dissipative scales.

The dimensionality of the system clearly exhibits a scale-dependent behavior characterized by an increase in the values of $D_{2}\left(\tau \right)$ with the mean frequency, approaching from a constant value $D_{2}\left(\tau \right)∼$ 2.7 for $f>f_{b}=0.4$ Hz. This suggests that magnetic field fluctuations are characterized by a superposition of processes working on different timescales and with different dimensionality. While MHD processes can be described by using a low-dimensional dynamical system, since $D_{2}<$ 2, the kinetic/dissipative domain dynamics cannot be represented as a linear system since at least three system variables ($D_{2}>$ 2) are needed to describe processes (perhaps dissipation) occurring at these scales.

An interesting result is the continuous change of the correlation dimension moving from MHD/inertial to kinetic/dissipative domains, which suggests that a single correlation dimension is not capable of describing the complexity features of solar wind magnetic field fluctuations at the MHD/inertial scales, while a single correlation dimension seems to describe kinetic features. This can be interpreted as the signature of the intermittent nature of fluctuations in the MHD/inertial domain, where a hierarchy of dimensions is necessary to describe the complex nature of the nonlinear energy cascade. Conversely, at the kinetic/dissipative scales where dissipation may occur, the correlation dimension seems to converge to a single value of $D_{2}∼$ 2.7. This is the temporal counterpart of the multifractal nature of turbulence in the MHD/inertial domain and of the monofractal nature of the dissipative regime, as also shown in Section 4.

We can characterize a dynamical system by looking at its phase-space dynamics in order to recover the existence of fixed points and their nature, as well as to investigate the presence of (strange) attractors [57]. Since by using the EMD we are able to decompose our time series into oscillating functions [48,59,60,61], we choose to reconstruct empirical modes according to the different dynamical regimes investigated. We can investigate the dynamics at the MHD/inertial and kinetic/dissipative scales in a separate way by defining

(20) $$\begin{array}{ccc} R_{I}\left(t\right) & = & \sum_{f_{k}\in f_{I}}c_{k}\left(t\right) \end{array}$$

(21) $$\begin{array}{ccc} R_{D}\left(t\right) & = & \sum_{f_{k}\in f_{D}}c_{k}\left(t\right) \end{array}$$

as the reconstructions of empirical modes with characteristic mean frequencies inside the intertial ($f_{I}$) and kinetic/dissipative ($f_{D}$) domains. In detail,

(22) $$\begin{array}{ccc} f_{I} & ≐ & \left\{f_{k}\;|\;10^{-3}\mathrm{Hz}<f_{k}<f_{b}\right\} \end{array}$$

(23) $$\begin{array}{ccc} f_{D} & ≐ & \left\{f_{k}\;|\;f_{k}>f_{b}\right\} \end{array}$$

being $f_{b}=0.4$ Hz.

**Figure 6:**
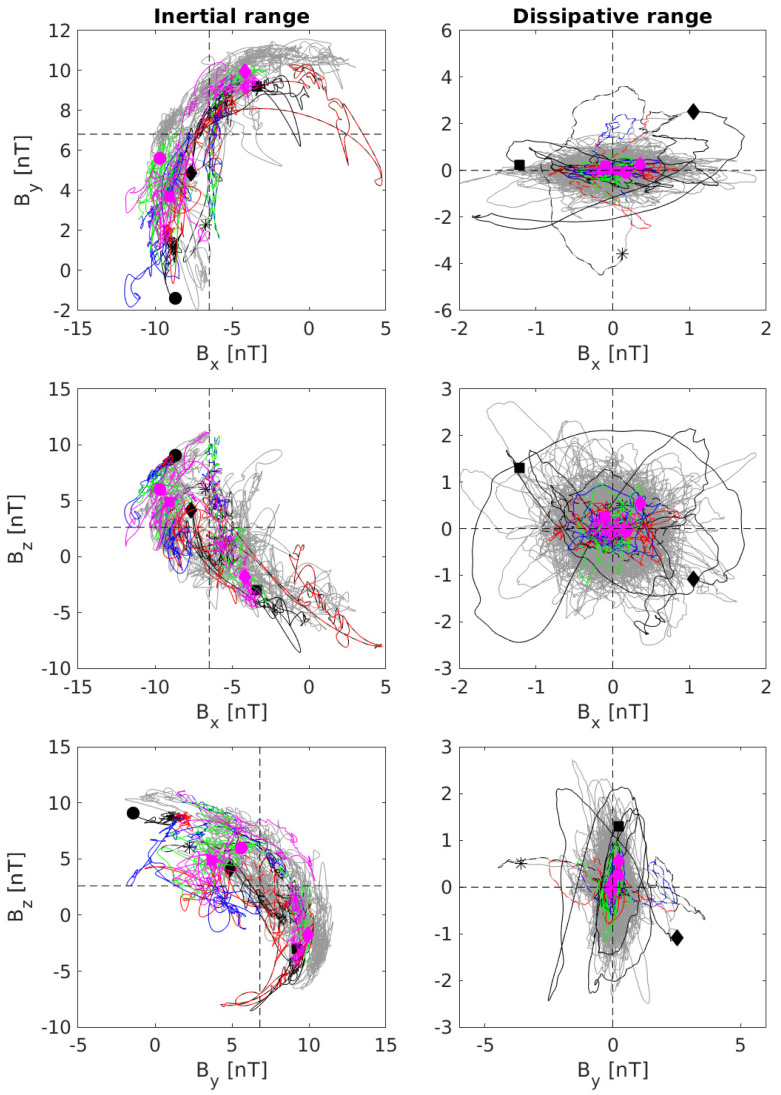
Phase-space portraits for the MHD/inertial range dynamics (**left panels**) and for the kinetic/dissipative range one (**right panels**). Symbols mark different phase-space trajectories with colors corresponding to different time instants (each trajectory starts with a black symbol and ends with a magenta one).

Figure 6 reports the phase-space portraits for the two different dynamical regimes, i.e., inertial scales (left panels) and kinetic/dissipative ones (right panels). The different symbols identify different phase-space trajectories starting at different phase-space positions, identified by a black symbol, and ending with a magenta one. The results look quite interesting and can be interpreted in dynamical system framework.

The dynamics seems to be characterized by an unstable orbit at inertial scales, so that the associated fixed point can be classified as a saddle. Indeed, starting from different phase-space positions each trajectory moves along an unstable manifold such that the system will approach the (unstable) fixed point being repelled on different (and opposite) phase-space points. Thus, the set is a repeller. This hyperbolic equilibrium point does not have any center manifolds, and, near it, the orbits of the system resemble hyperbolas. Conversely, the dynamics at kinetic/dissipative scales is characterized by a set which is an attractor since all phase-space trajectories tend to move towards the stable fixed point, which can be identified as a node. This fixed point, due to its fractal dimension and structure (see Section 4), is a chaotic strange attractor, extremely sensitive to initial conditions. By considering two arbitrarily close initial phase-space positions near the attractor, after several time steps they will move on phase-space positions far apart, and after other several time iterations will lead to phase-space positions which are arbitrarily close together. Thus, the dynamics never depart from the attractor [56].

**Figure 7:**
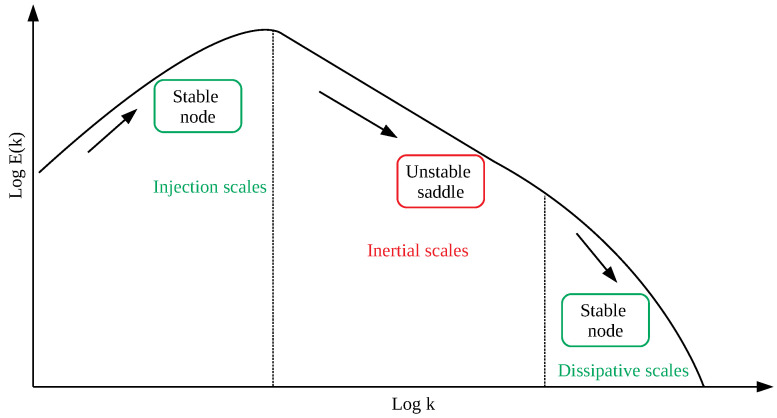
A sketch of the different dynamical regimes.

The obtained results seems to suggest a new view of the dynamics of the solar wind at different scales from the MHD/inertial domain down to the kinetic/dissipative one. The system undergoes a saddle-node bifurcation, a local bifurcation in which two fixed points collide and annihilate each other, with an unstable fixed point (saddle) and a stable one (node). This means that both the inertial and kinetic/dissipative ranges can be seen as fixed points of the governing system equations, one unstable and the other stable. In this way, the phenomenological model of the Richardson cascade [24,26,28,29] can be interpreted in the different context of the dynamical system theory. Energy is injected at a scale *L*, which represents a stable fixed point of the system; then, when nonlinear interactions develop, corresponding to changes in one or more dynamical bifurcation parameters, the dynamics of the system changes, moving towards an unstable fixed point (i.e., the inertial regime) which, due to its repeller nature, forces the system to explore the available phase-space until a stable fixed point (i.e., the kinetic/dissipative domain) is reached (Figure 7). In a simple conceptual model, a bifurcation parameter could be the timescale of the different processes operating inside the MHD/inertial and kinetic/dissipative domains such that the dynamics of the system, represented in our case by the magnetic field components $B_{i}$, can be seen as solely dependent on τ

(24) $$\begin{array}{c} {\dot{B}}_{i}=g\left(B_{i},B_{j},\tau \right). \end{array}$$

The system is surely characterized by a chaotic dynamics due its dimension (our system is described by three variables) with nonlinear interactions between the different variables (i.e., $g(B_{i},B_{j},\tau )$ is a nonlinear function of $B_{i},B_{j}$) as required when describing turbulent features (e.g., [1,24,26]).

## 6. Conclusions

Solar wind magnetic field fluctuations at different scales have been investigated by employing both a multifractal and a chaotic approach. The multifractal analysis has been performed by using a novel formalism, the EMD-based dominant amplitude multifractal formalism, through which increments are derived by using local properties of fluctuations at different scales obtained by using the Empirical Mode Decomposition method. The results suggest that MHD fluctuations show an intermittent character, well described in the framework of classical multifractal models (like the *p*-model (e.g., [26])); conversely, magnetic field fluctuations at kinetic scales (i.e., beyond the ion inertial length) show a monofractal behavior, in agreement with previous findings (e.g., [52,53,54]).

The phase-space dynamics of the two ranges of scales, i.e., inside the MHD/inertial and kinetic/dissipative domains, is characterized by a different degree of chaos, because the system is more chaotic when moving from the MHD down to the kinetic scales. An unstable manifold is recovered for the MHD scales, characterizing an unstable saddle for the magnetic field dynamics. Conversely, a stable manifold, corresponding to a stable node, is found at kinetic scales, suggesting the occurrence of a saddle-node bifurcation passing from MHD down to kinetic scales. These results can open the way to new perspectives in approaching scale-to-scale dynamics of solar wind magnetic field fluctuations as well as in deriving conceptual models to explain the observed dynamical regimes.

## Abbreviations

The following abbreviations are used in this manuscript:

DS	Degree of Stationarity
EMD	Empirical Mode Decomposition
EMD-DAMF	Empirical Mode Decomposition-Dominant Amplitude Multifractal Formalism
ESA	European Space Agency
FGM	Fluxgate Magnetometer
GSE	Geocentric Solar Ecliptic
HSA	Hilbert Spectral Analysis
HT	Hilbert Transform
IMF	Intrinsic Mode Function
KAW	Kinetic Alfvén Wave
MHD	Magnetohydrodynamics
STAFF	Spatio Temporal Analysis of Field Fluctuations
WTMM	Wavelet Transform Modulus Maxima
